# Mammalian Sterile 20-Like Kinase 1 Mediates Neuropathic Pain Associated with Its Effects on Regulating Mitophagy in Schwann Cells

**DOI:** 10.1155/2022/3458283

**Published:** 2022-05-24

**Authors:** Zi Huang, Pei-Yao Xiao, Jing-Yan Chen, Qing Zeng, Bei-Xu Huang, Jian Yu, Song-Jie Liao

**Affiliations:** ^1^Department of Neurology, The First Affiliated Hospital, Sun Yat-sen University, No. 58 Zhongshan Road 2, Guangzhou 510080, China; ^2^Neurological Diseases, National key Clinical Department and Key Discipline of Neurology, No. 58 Zhongshan Road 2, Guangzhou 510080, China; ^3^Guangzhou First People's Hospital, No. 1 Panfu Road, Guangzhou 510000, China

## Abstract

Myelin degradation initiated by Schwann cells (SCs) after nerve injury is connected to the induction and chronicity of neuropathic pain (NP). Mitophagy, a selective clearance of damaged mitochondria via autophagy, contributes to the maintenance of normal function in SCs. Mitochondrial function and mitophagy activity are highly modulated by mammalian ste20-like kinase1 (Mst1). However, whether Mst1 can regulate mitophagy in SCs to play a role in NP remains poorly understood. In the present study, Sprague-Dawley rats were subjected to chronic constriction injury (CCI) on the sciatic nerve to induce NP. Small interfering RNA of Mst1 was applied to the injured sciatic nerve to knockdown Mst1. Behavioral tests were performed to evaluate NP, and myelin degeneration was assessed by transmission electron microscope and immunofluorescence. Autophagy and mitophagy were detected in the injured sciatic nerve and cultured SCs (RSC96 cells) by Western blot. ROS level, mitochondria membrane potential, and apoptosis were assessed in vitro via flow cytometry and Western blot. Mst1 knockdown alleviated mechanical allodynia and thermal hyperalgesia in the CCI-induced NP model and rescued myelin degeneration of the injured nerve. Meanwhile, CCI-increased levels of Parkin and p62 were reversed by Mst1 knockdown. In vitro RSC96 cells were subjected to starvation to induce mitophagy. Protein levels of mitochondrial Parkin and mitochondrial p62 significantly increased after Mst1 knockdown, while those in the cytosol diminished indicate that the translocation of Parkin and p62 from the cytosol to the mitochondria was promoted by the knockdown of Mst1. In addition, Mst1 knockdown reduced ROS level and apoptosis activity, while enhancing mitochondria membrane potential in RSC96 cells. The study showed that Mst1 knockdown alleviated CCI-induced NP, associated with enhanced Parkin recruitment to mitochondria and subsequent mitophagy degradation, thus preserving mitochondrial function and myelin integrity.

## 1. Introduction

International Association for the Study of Pain (IASP) redefined neuropathic pain (NP) as pain caused by diseases or lesions of the somatosensory system in 2012. NP has a prevalence rate of 0.9% to 17.9% suggested by different studies [1] and imposes a heavy burden on patients and society. Current NP therapies inhibiting aberrant neuronal activity provide limited efficacy and could cause unavoidable side effects [2]. Fortunately, the identification of Schwann cells (SCs) in NP casts light on the etiology of NP and might culminate in methods for its treatment [3–5].

SCs undergo morphological and functional changes after nerve injury [6–8] which is deemed as an important pathogenesis in the development of NP. In the peripheral nerve, mitochondria in SCs are critical in maintaining axo-glial interaction to support axons and sustain normal peripheral nerve function [9]. Mitochondrial quality control is achieved by the selective clearance of damaged mitochondria via mitophagy. Impaired mitophagy in the peripheral nerve leads to an accumulation of dysfunctional mitochondria, causing excessive reactive oxygen and microglial activation, as a result, myelinoclasis and NP [10].

Mammalian ste20-like kinase1 (Mst1) is a critical serine/threonine kinase in Hippo pathway, which is involved in cell proliferation, survival, mobility, stemness, and differentiation [11]. Inhibition to the downstream effectors of Mst1 in Hippo pathway-YAP/TAZ in the spinal cord alleviates CCI-induced symptoms of NP [12]. Several lines of evidences have identified Mst1 as an upstream mediator of mitophagy. Parkin is recruited to the depolarized mitochondrial outer membrane (MOM) and mediates mitophagy as a ubiquitin ligase. Parkin-dependent mitophagy activity is enhanced after Mst1 inhibition, and this confers protection against diabetic cardiomyopathy, liver fatty disease, and septic cardiomyopathy [13–15]. However, whether Mst1 is involved in NP has not been revealed by now. In the present study, experiments were performed to investigate the involvement of Mst1 in NP and to uncover the mechanisms underlying Mst1-mediated regulation on mitophagy in SCs, which is critical in the process of NP.

## 2. Materials and Methods

### 2.1. CCI of Rats and In Vivo Knockdown of Mst1

Adult male Sprague-Dawley rats were purchased from the Beijing Vital River Laboratory Animal Technology Co., Ltd. with an initial weight of 280-350 g. They were maintained under specific-pathogen-free and light-cycled system with no restriction on food and water in Animal Care Facility of Sun Yat-sen University. The protocol was approved by the Animal Care Committee, Sun Yat-sen University, China (SYSU-IACUC-2020-000256). The experiments were performed following the Animal Welfare Act Guide for Use. The procedures were conducted and modified according to previous studies [16, 17]. Rats were anesthetized with sodium pentobarbital (50 mg/kg, i.p., supplemented if necessary). An incision was made at the level of the middle of the thigh and then the common sciatic nerve was exposed by blunt dissection through the biceps femoris. About 1 cm of the sciatic nerve was freed proximal to the trifurcation, and 3 ligatures (4-0 chromic gut) were tied loosely around the freed nerve with about 1 mm spacing. PF-127 (Sigma-Aldrich, St. Louis, MO, USA) was dissolved in 0.1 M phosphate-buffered saline (PBS, pH 7.6) to achieve a gel with a final concentration of 25% (*m*/*v*). The mixture was shaken overnight at 4°C until complete dissolution and then filtered (0.22 *μ*m aperture) and stored at 4°C for further use. The small interfering RNA (siRNA) for in vivo use was designed by RiboBio (Guangzhou RiboBio Co., Ltd, China) with the sequence 5′-CTCCGAAACAAGACGTTAA-3′. Rats were randomly divided into 4 groups: sham group, CCI group, CCI+si-NC group (120 *μ*l PF-127 gel+4 nmol siRNA of negative control), and CCI+si-Mst1 (120 *μ*l PF-127 gel+4 nmol siRNA of Mst1). The sham group received a sham procedure (the sciatic nerve was exposed without ligation). In the CCI+si-NC group and the CCI+si-Mst1 group, a 120 *μ*l mixture of gel and siRNA was dripped surrounding the sciatic nerve immediately after the ligation. At last, the incision was closed in layers.

### 2.2. Behavioral Tests

To measure the paw withdrawal threshold (PWT) [18], rats were placed individually in a plastic chamber with a metal mesh bottom. Behavioral acclimation was allowed for about 20 minutes prior the test, until cage exploration and major grooming activities ceased. One of a series of 9 von Frey hairs with logarithmically incremental stiffness (0.40, 0.60, 1.0, 2.0, 4.0, 6.0, 8.0, 10.0, and 15.0 g) was presented perpendicular to the plantar surface of the left hind paw. Stimuli were presented with appropriate force to cause slight buckling against the left hind paw and held for approximately 6-8 seconds at intervals of at least 15 seconds. Sharp paw withdrawal or paw licking during stimulation was noted as positive responses. Ambulation was considered an ambiguous response, and in such case, the stimulus was repeated. Testing was initiated with the 2.0 g hair. Stimuli were presented in a consecutively ascending or descending fashion. In the presence of a positive response, a next weaker stimulus was given; in the event of a negative response, a next stronger stimulus was chosen. Four additional responses were subsequently recorded after the response threshold crossed for the first time. In cases where continuously positive or negative responses were observed to the exhaustion of the stimuli set, a stimulus of 15.00 g or 0.40 g was assigned, respectively. Data were inputted into a program called CellG to calculate the PWT.

A radiant thermal stimulator (IITC 390) was applied to measure the paw withdrawal latency (PWL) [19]. Rats were placed individually in plexiglass compartments on an elevated glass platform. A light thermal stimulator was placed under the platform and focused on the plantar surface of left hind paw. The time required for sharp paw withdrawal or paw flicking was recorded. The radiant heat intensity was adjusted until the basal PWL for the normal hind paw was between 10 and 15 seconds. A cutoff of 20 seconds was used to avoid tissue damage. Each animal was tested for 5 times with an interval of 5 minutes. The average of 3 middle values was noted as PWL.

The behavior tests were performed one day before the surgery as base line and every 2 or 3 days during 14 postoperative days to evaluate mechanical allodynia and thermal hyperalgesia, respectively.

### 2.3. Western Blot

On the 4^th^ postoperative day, rats were anesthetized with sodium pentobarbital and perfused transcardially with 0.9% saline. The left sciatic nerve segment was excised carefully from 0.5 cm proximal to 0.5 cm distal to the lesion. Sciatic nerve was snap-frozen in liquid nitrogen for several minutes and stored at -80°C. RIPA lysis buffer (Thermo, MA, United States) containing a protease inhibitor was used for sample homogenization. The protein concentration was measured using BCA protein assay kit (Thermo, MA, United States) as instructed. After denaturation at 95°C for 5 minutes, protein samples were stored at -80°C for further use. Equal amounts of protein (30 *μ*g/well) were separated on 12.5% SDS-polyacrylamide gel electrophoresis (PAGE) (Bio-Rad) and then transferred into the polyvinylidene fluoride (PVDF) membranes (Merck Millipore). For blocking, PVDF membranes were treated with 5% nonfat dry milk at room temperature for 1 hour. Then primary antibodies, including Mst1 (ab51134, 1 : 10000; Abcam), Parkin (NBP2-67017, 1 : 1000; Novus Biologicals), p62 (5114S, 1 : 1000, Cell Signaling Technology), LC3 (L8918, 1 : 1000, Cell Signaling Technology), Tom20 (sc-17764, 1 : 1000; Santa Cruz Biotechnology), GAPDH (5174S, 1 : 1000; Cell Signaling Technology), COX-IV (4850 T, 1 : 1000; Cell Signaling Technology), Caspase9 (ab184786, 1 : 1000; Abcam), Cytochrome-c (Cyt-c) (ab133504, 1 : 1000, Abcam), and Bax (ab32503, 1 : 1000, Abcam) were diluted in TBST containing 5% nonfat milk, added to the membrane, and incubated at 4°C overnight. After being washed with TBST for 3 times, the membranes were incubated with HRP-conjugated secondary antibodies (Cell Signaling Technology) for 1 hour at room temperature. The reaction was detected using immobilon western chemilum HRP substrate (Merck Millipore). The bands were visualized using Asherman Imager 600 and quantified by ImageJ software (National Institutes of Health, Bethesda, MD).

### 2.4. Immunofluorescence

On the 14^th^ postoperative day, rats were anesthetized with sodium pentobarbital and perfused transcardially with 0.9% saline. The left sciatic nerve segment was excised carefully and fixed in 4% paraformaldehyde for 6 h, subsequently dehydrated in 20% and 30% sucrose at 4°C overnight, and embedded in OCT. After slicing, sections were air-dried overnight and stored at -40°C. The frozen sections were equilibrated to room temperature for 10 min and washed in 0.01 M PBS for 10 min, then treated with citrate buffer at 95°C for 5 min and cooled to room temperature. The tissue sections were blocked with immunofluorescence blocking solution containing 0.3% Triton X-100 for 1 hour at room temperature. Afterwards, the sections were incubated with primary antibodies against S100 (ab868, 1 : 200; Abcam) and P0 (ab31851, 1 : 100; Abcam) overnight at 4°C, washed, and then incubated with fluophore-conjugated secondary antibodies (Cell Signaling Technology) for 1 hour at room temperature. Tissues were covered with mounting medium including 4′,6-diamidin-2-phenylindol solution (DAPI) and imaged by a fluorescence microscope (Nikon DS-Ri2).

### 2.5. Transmission Electron Microscope (TEM)

The animals were anesthetized with pentobarbital sodium and perfused transcardially with 0.9% saline 14 days after surgery. The injured nerve was rapidly removed and fixed in glutaraldehyde (2.5% in 0.1 M PBS, pH 7.6). Then, the tissue was washed, postfixed in aqueous 1% osmium tetroxide for 2 hours, dehydrated in ethanol with a concentration gradient, embedded in Epson, and stained with lead citrate and uranyl acetate. Finally, the specimen was examined using a Hitachi H600 TEM. ImageJ analysis software was used to measure the diameter of axons and the outer diameter of each myelinated nerve fiber. G-ratio (only for the myelinated axons) value was calculated using the following formula: G‐ratio = axon diameter/outer myelin diameter.

### 2.6. Schwann Cell Culture and Transfection

RSC96 cells were purchased from the Institute of Cell Research, Shanghai Academy of Sciences. The cells were cultured in Dulbecco's modified Eagle medium (DMEM) (Thermo, MA, United States) containing 10% fetal bovine serum (FBS) (Thermo, MA, United States) and humidified atmosphere of 5% CO_2_ at 37°C. For starvation, cells were gently washed twice with PBS and cultured in DMEM with 1% FBS for 24-h. RSC96 cells were transfected with Mst1 siRNA or scrambled siRNA as control, respectively, using Lipofectamine 3000 (Thermo, MA, United States) according to the manufacturer's instructions. The sequence of Mst1 siRNA was as follows: 5′-CTCCGAAACAAGACGTTAA-3′.

### 2.7. Mitochondrial Extraction

Mitochondria and cytosol were isolated using a commercial mitochondria isolation kit (Thermo, MA, United States) and the protocol was followed as the manufacturer's instruction. Cell pellet was homogenized in Mitochondria Isolation Reagent A containing protease inhibitor. After being ground with a glass homogenizer, the sample was centrifuged at 700 g for 10 minutes at 4°C. The supernatant was collected in another tube and centrifuged at 3,000 g for 15 minutes at 4°C to obtain a more purified fraction of mitochondria. The supernatant was transferred to a new tube (cytosol fraction). The pellet was dissolved in Mitochondria Isolation Reagent C and centrifuged at 12,000 g for 5 minutes to obtain the mitochondrial pellet on ice.

### 2.8. ROS Detection

The level of ROS was detected by DCFH-DA staining (Sigma-Aldrich, St. Louis, MO, USA). Cells were incubated in serum-free DMEM containing 10 *μ*M DCFH-DA for 20 min, shielded from light. After being washed twice with PBS, the fluorescence intensity was measured by flow cytometry on Cytoflex.

### 2.9. Mitochondrial Membrane Potential (MMP) Detection

MMP was determined by the fluorescence change of JC-1 Mitochondrial Membrane Potential Dye (Thermo, MA, United States). After being washed twice with PBS, cells were stained by 200 *μ*M JC-1 Mitochondrial Membrane Potential Dye in serum-free DMEM for 20 min, shielded from light. Subsequently, the fluorescence intensity was measured by Cytoflex. JC-1 accumulates in mitochondria in a potential-dependent way, suggested by a fluorescence emission shift from green (~529 nm) to red (~590 nm) when MMP increases. Consequently, mitochondrial depolarization is indicated by a decreased ratio of red to green fluorescence intensity.

### 2.10. Statistical Analysis

All statistical analyses were performed with IBM SPSS Statistics for Windows, version 20.0 (IBM Corp., Armonk, NY, USA). Data were presented as the mean ± SD. Two-way repeated measures ANOVA, followed by Bonferroni's post hoc test, was used to assess PWL and PWT among different groups. Student's *t*-test was applied to compare data between two different groups. One-way ANOVA, followed by Bonferroni's post hoc test, was used to analyze other quantitative data. A two-tailed *P* value < 0.05 was considered statistically significant.

## 3. Results

### 3.1. Mst1 Knockdown Alleviated CCI-Induced Heat Hyperalgesia and Mechanical Allodynia

**Figure 1:**
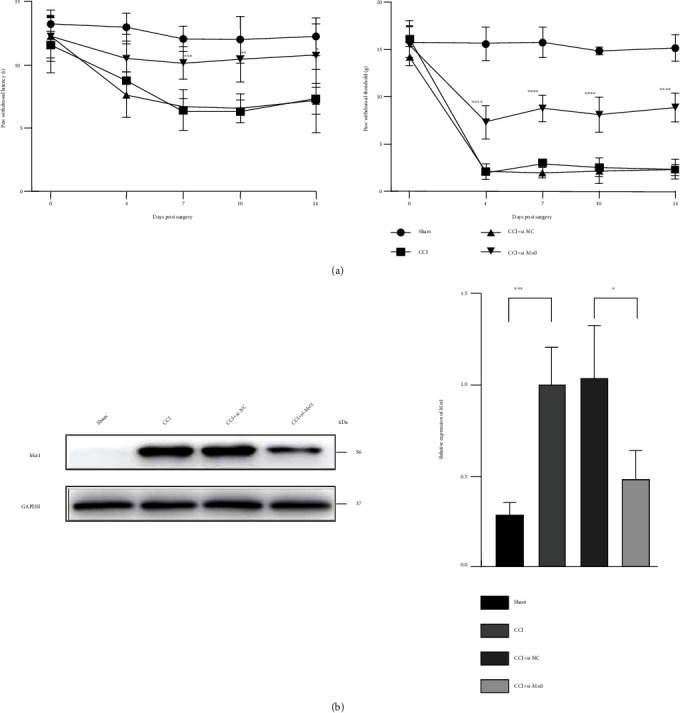
Mst1 knockdown alleviated CCI-induced heat hyperalgesia and mechanical allodynia in rats. (a) Quantitative analysis of PWL (s) and PWT (g). ^∗^*P* < 0.05,  ^∗∗^*P* < 0.01,  ^∗∗∗^*P* < 0.001, and^∗∗∗∗^*P* < 0.0001, vs the CCI+si-NC group at the same time point; *n* = 6. Statistical significance was determined by two-way repeated measures ANOVA, followed by Bonferroni's post hoc test. (b) Protein expression of Mst1 with GAPDH as a loading control. ^∗^*P* < 0.05 and^∗∗∗^*P* < 0.001; *n* = 4. Statistical significance was determined by one-way ANOVA, followed by Bonferroni's post hoc test. All data were expressed as the mean ± SD. CCI+si-NC: injured sciatic nerves were treated with scrambled siRNA as negative control; CCI+si-Mst1: injured sciatic nerves were treated with Mst1 siRNA.

CCI on the sciatic nerve causes heat hyperalgesia and mechanical allodynia shown by decreased PWL and PWT in rats, both of which are the typical symptoms of NP [20]. The effects of CCI started at 4 days and lasted for at least 14 days after operation when compared with the sham group (Figure 1(a)). Simultaneously, CCI also augmented the expression of Mst1 protein on the sciatic nerve at 4 days after surgery (Figure 1(b)). By contrast, Mst1 knockdown by the application of Mst1-siRNA to the injured sciatic nerve, which was confirmed by Western blot, significantly increased PWL and PWT at different time points (Figures 1(a) and 1(b)). Together, these results showed that Mst1 contributed to CCI-induced heat hyperalgesia and mechanical allodynia.

### 3.2. Mst1 Knockdown Protected the Sciatic Nerve from CCI-Induced Myelin Degeneration

**Figure 2:**
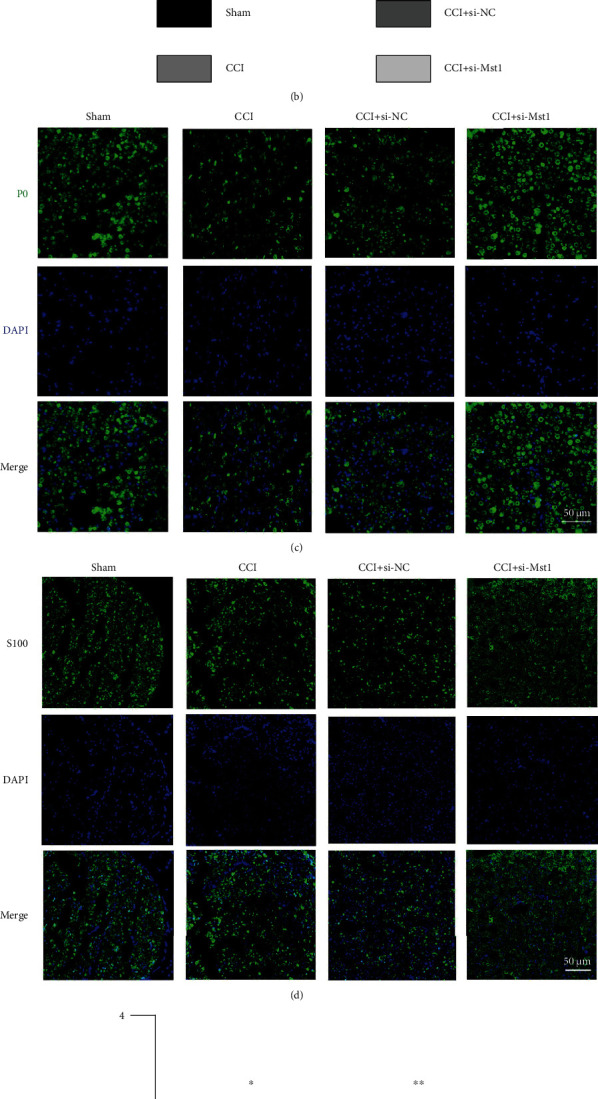
Mst1 knockdown protected the sciatic nerve from CCI-induced myelin degeneration. (a) TEM: representative images of the sciatic nerve. Black arrows point to myelin and stars refer to axon. 5800x. Scale bar: 5 *μ*m. (b) Quantitative analysis of G-ratio. ^∗^*P* < 0.05 and^∗∗∗^*P* < 0.001; *n* = 3. Statistical significance was determined by one-way ANOVA, followed by Bonferroni's post hoc test. All data were expressed as the mean ± SD. (c) Representative images of P0 staining. 400x. Scale bar: 50 *μ*m. (d) Representative images of S100 staining. 400x. Scale bar: 50 *μ*m. (e) Quantitative analysis of the number of S100-positive cells per sciatic nerve. ^∗^*P* < 0.05 and^∗∗^*P* < 0.01; *n* = 3. Statistical significance was determined by one-way ANOVA, followed by Bonferroni's post hoc test. All data were expressed as the mean ± SD. CCI+si-NC: injured sciatic nerves were treated with scrambled siRNA as negative control; CCI+si-Mst1: injured sciatic nerves were treated with Mst1 siRNA.

To verify whether Mst1 knockdown alleviated pain behavior in response to thermal and mechanical stimuli via myelin protection in rats, we applied TEM and immunofluorescence analysis with antibodies against P0 (a major component in myelin membrane determining myelin thickness) and S100 (a specific marker of SCs indicating cell migration and progression) on sections derived from the injured sciatic nerve to evaluate morphological changes of myelin. TEM showed that in the sham group, the membrane of SCs wrapped around and encapsulated the axon to form a compact multilamellar myelin sheath (Figure 2(a)). CCI induced evident degradation and structural collapse of myelin in the sciatic nerve, accompanied with axonal degeneration under TEM as well as myelin derangement and attenuation by P0 staining (Figures 2(a) and 2(c)). G-ratio, a ratio of the inner to the outer radius of myelin sheath under TEM, has been shown to be negatively correlated with myelin thickness [21]. The analysis of G-ratio suggested that CCI caused a reduction of myelin thickness, which was rescued by Mst1 knockdown (Figure 2(b)). The beneficial effect of Mst1 knockdown on myelin was also proved by the relatively preserved myelin morphology and increased number of S100-positive SCs observed in the CCI+si-Mst1 group compared with the CCI+si-NC group (Figure 2).

### 3.3. Mst1 Knockdown Improved CCI-Induced Impairment to Mitophagy Degradation

**Figure 3:**
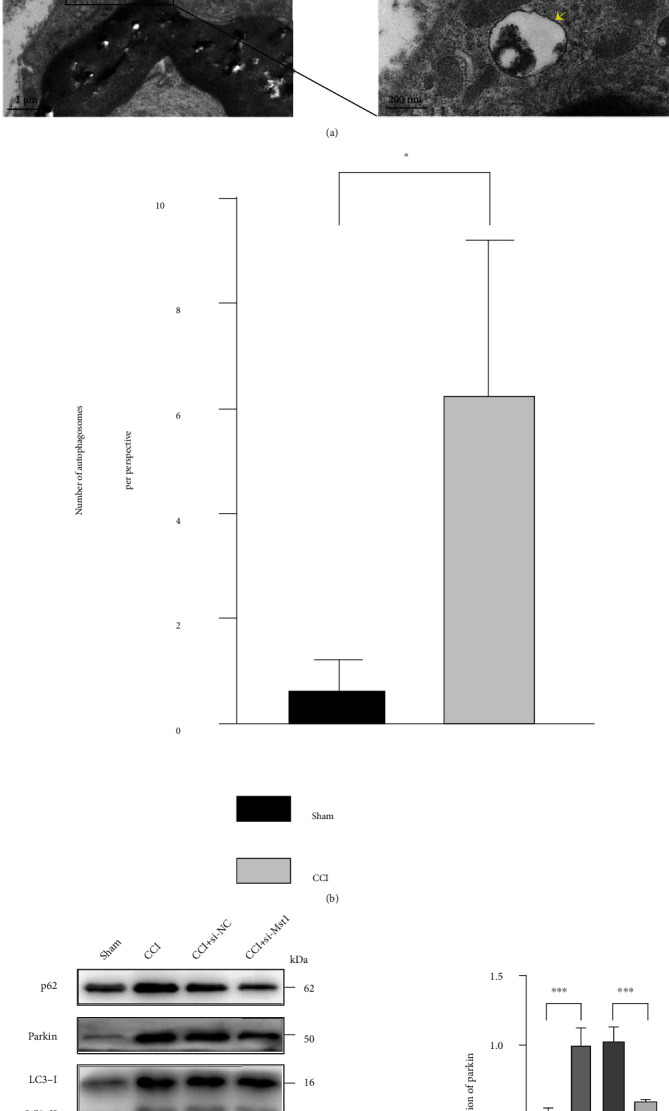
Mst1 knockdown might promote autophagic influx and affect Parkin-dependent mitophagy impaired by CCI. (a) TEM analysis of autophagy and mitophagy in injured sciatic nerves in the sham and the CCI groups. The right pictures are the enlarged view of box areas in the left pictures. Red arrows point to autophagosomes. White pentagrams indicate the engulfed mitochondria and the remnant of mitochondrial structure. Yellow arrows refer to the autolysosome with digested mitochondria. Red pentagrams indicate the SCs. Left pictures: 5800x; scale bar: 1 *μ*m. Right pictures: 37000x; scale bar: 200 nm. (b) Quantitative analysis of the number of autophagosomes per perspective at 5800x magnification in the sham and the CCI group. ^∗^*P* < 0.05; *n* = 3. Statistical significance was determined by Student's *t*-test. All data were expressed as the mean ± SD. (c) Protein expression of Parkin, p62, and LC3-II with GAPDH as a loading control, and relative level of LC3-II/I ratio. ^∗∗^*P* < 0.01,  ^∗∗∗^*P* < 0.001, and^∗∗∗∗^*P* < 0.0001; ^#^*P* < 0.05 vs. other groups; *n* = 4. Statistical significance was determined by one-way ANOVA, followed by Bonferroni's post hoc test. All data were expressed as the mean ± SD. CCI+si-NC: injured sciatic nerves were treated with scrambled siRNA as negative control; CCI+si-Mst1: injured sciatic nerves were treated with Mst1 siRNA.

We applied TEM and Western blot to further investigate whether Mst1 had the potential to mediate NP via mitophagy. TEM analysis of the injured sciatic nerve in the CCI group revealed abundant engulfment of mitochondria by autophagosomes, as well as autolysosomes with digested mitochondria-like structures inside SCs (Figures 3(a) and 3(b)). These were barely observed in the sham group (Figures 3(a) and 3(b)). In parallel with this, the expression of LC3-II was overtly increased in the CCI group (Figure 3(c)). These results indicated that CCI activated mitophagy/autophagy in the injured sciatic nerve. Meanwhile, the degradation of mitophagy/autophagy was impaired after CCI, as evidenced by increased protein levels of Parkin and p62 in the CCI group (Figure 3(c)). Mst1 knockdown significantly counteracted CCI-induced impairment to mitophagy degradation (Figure 3(c)). Taken together, Mst1 knockdown might promote the successful clearance of accumulated mitophagy/autophagy substrates caused by CCI, which was further investigated in the following in vitro study.

### 3.4. Mst1 Knockdown Promoted Parkin Recruitment to Mitochondria in SCs

**Figure 4:**
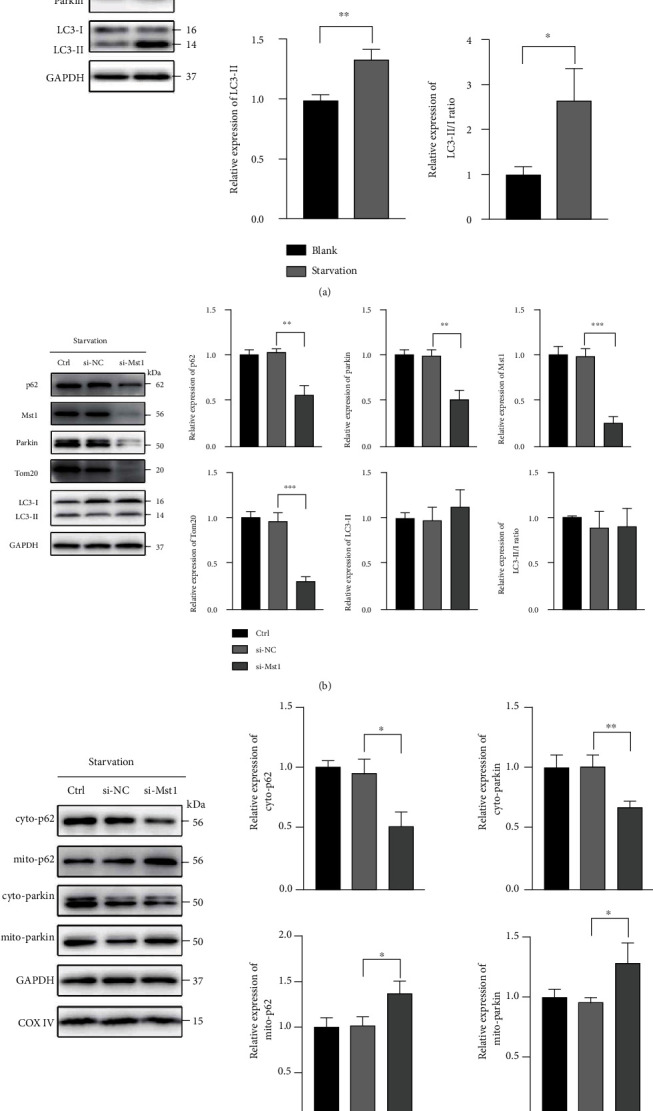
Mst1 knockdown promoted Parkin translocation to mitochondria in cultured SCs. (a) Protein expression of Mst1, Parkin, and LC3-II with GAPDH as a loading control and relative level of LC3-II/I ratio. ^∗^*P* < 0.05,  ^∗∗^*P* < 0.01, and^∗∗∗^*P* < 0.001; *n* = 3. Statistical significance was determined by Student's *t*-test. Blank group: cells were cultured in DMEM with 10% FBS for 24-h. Starvation group: cells were cultured in DMEM with 1% FBS for 24-h. (b) Protein expression of p62, Mst1, Parkin, Tom20, and LC3-II with GAPDH as a loading control and relative level of LC3-II/I ratio. ^∗∗^*P* < 0.01 and^∗∗∗^*P* < 0.001; *n* = 3. Statistical significance was determined by one-way ANOVA, followed by Bonferroni's post hoc test. (c) Protein expression of cyto-p62, mito-p62, cyto-Parkin, and mito-Parkin, with GAPDH as a loading control for proteins in the cytosol and COX-IV as a loading control for mitochondrial proteins. ^∗^*P* < 0.05 and^∗∗^*P* < 0.01; *n* = 3. Statistical significance was determined by one-way ANOVA, followed by Bonferroni's post hoc test. All data were expressed as the mean ± SD. Control group: no treatment. si-NC group: cells were treated with scrambled si-RNA as negative control. si-Mst1 group: cells were treated with Mst1 siRNA. 24 h after transfection, all groups were cultured in DMEM with 1% FBS for another 24 h.

The effects of Mst1 knockdown on autophagy and Parkin-dependent mitophagy were investigated by using a Schwann cell line (RSC96 cells). Firstly, we investigated the effect of Mst1 on mitophagy in RSC96 cells subjected to starvation. Starvation augmented the expression of Mst1, Parkin, and LC3-II (Figure 4(a)). Mst1 knockdown diminished starvation-induced expression of Parkin as well as p62 and Tom20 (MOM marker) but had no effect on the expression of LC3-II (Figure 4(b)). Subsequently, the mitochondria and cytosol fractions were separately isolated and analyzed. Mst1 knockdown increased the protein levels of mitochondrial Parkin and p62 but reduced those in the cytosol (Figure 4(c)). Taken together, these results indicated that Mst1 knockdown promoted mitophagy via an enhancement of Parkin recruitment from cytosol to mitochondria, leading to mitochondrial clearance.

### 3.5. Mst1 Knockdown Sustained Mitochondrial Function and Prevented Mitochondrial Apoptosis

**Figure 5:**
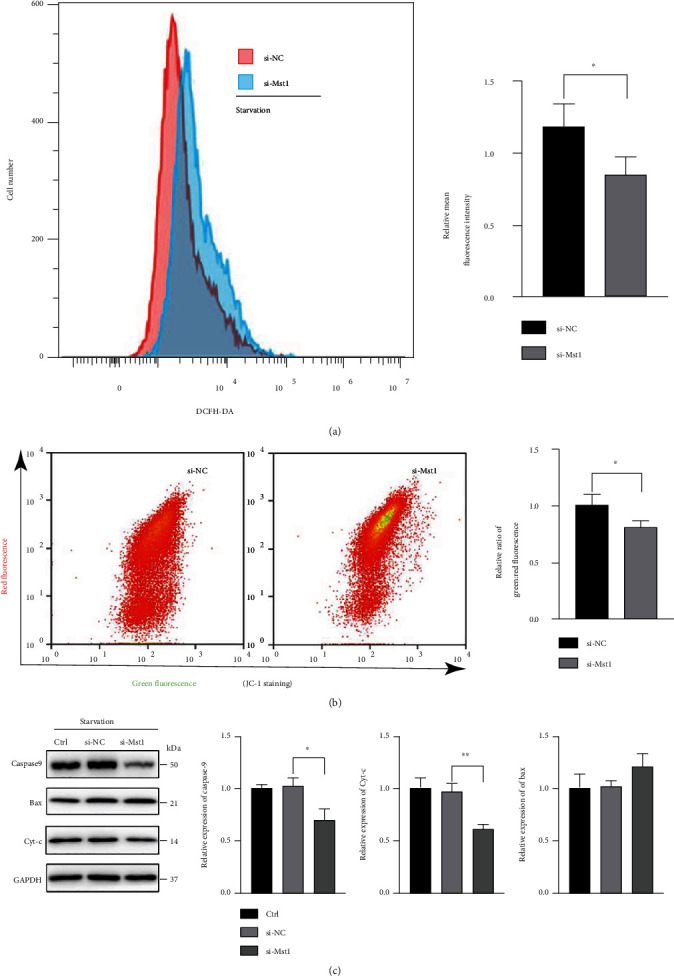
Mst1 knockdown sustained mitochondrial function and prevented mitochondria apoptosis. (a) Measurement and quantitative analysis of mean fluorescence intensity for DCFH-DA staining. ^∗^*P* < 0.05; *n* = 3. Statistical significance was determined by Student's *t*-test. (b) Measurement and quantitative analysis of JC-1 staining, expressed as ratio of green fluorescence intensity to red fluorescence intensity. ^∗^*P* < 0.05; *n* = 3. Statistical significance was determined by Student's *t*-test. si-NC group: cells were treated with scrambled si-RNA as negative control. si-Mst1 group: cells were treated with siRNA of Mst1. 24 h after transfection, all groups were cultured in DMEM with 1% FBS for another 24 h. (c) Protein expression of Caspase9, Bax, and Cyt-c, with GAPDH as loading control. ^∗^*P* < 0.05 and^∗∗^*P* < 0.01; *n* = 3. Control group: no treatment. si-NC group: cells were treated with scrambled si-RNA as negative control. si-Mst1 group: cells were treated with siRNA of Mst1. 24 h after transfection, all groups were cultured in DMEM with 1% FBS for another 24 h. Statistical significance was determined by one-way ANOVA, followed by Bonferroni's post hoc test. All data were expressed as the mean ± SD.

The effects of Mst1 on mitochondrial function were investigated in starved RSC96 cells. Mitochondria are the main source of ROS production, and mitophagy is a key process to eliminate ROS [22]. Consequently, cellular ROS level was determined by DCFH-DA staining as an indicator of mitochondrial status. Starved RSC96 cells subjected to Mst1 knockdown showed a notably reduced fluorescence intensity of DCFH-DA compared with the si-NC group (Figure 5(a)), indicating a decreased level of ROS. In addition, JC-1 immunofluorescence was used to monitor MMP. In defective mitochondria following damage or stress, the MMP decreased and JC-1 was monomer, resulting in enhanced green fluorescence. In starvation status, cells in the si-Mst1 group had lower green to red ratio compared with the si-NC group (Figure 5(b)), suggesting that MMP was enhanced and mitochondrial function was potentially reserved after Mst1 knockdown.

In addition, as mitochondria are also essential for apoptosis, mitochondria-related apoptosis was detected in starved RSC96 cells. Mst1 knockdown reduced the expression of mitochondrial proapoptotic proteins (Caspase9 and Cyt-c), but had no effect to mitochondrial antiapoptotic protein such as Bax (Figure 5(c)).

Altogether, these findings supported that Mst1 knockdown protected RSC96 cells against starvation insult, as evidenced by preserved mitochondrial function with reduced ROS level and apoptosis activity, as well as enhanced MMP.

## 4. Discussion

NP is a complex and sometimes recurrent disease that patients will seek help for, and it imposes heavy financial burden on the whole society [23]. Our results illustrated a crucial involvement of Mst1 in NP. Mst1 knockdown had the potential to alleviate NP via myelin protection, and this could be associated with its effects on mitophagy regulation. Mst1 knockdown promoted autophagic influx and regulated mitophagy in the injured sciatic nerves of rats. In Schwann cells, Mst1 knockdown enhanced mitophagy by promoting Parkin recruitment from cytosol to mitochondria and thus rescued mitochondrial function and prohibited apoptosis.

Mst1 was identified as a critical factor in the development of neurological diseases. In cerebral ischemia-reperfusion injury, Mst1-induced microglial activation contributed to neuronal death, and microglia-specific knockdown of Mst1 alleviated the stroke-related brain injury [24]. The upstream activator of Mst1, c-Abl tyrosine kinase, led to mitochondrial abnormality, excessive ROS production and neuronal apoptosis in prion diseases [25]. However, the role of Mst1 in NP remains unknown. We first established a CCI model on rats to simulate nerve injury-induced NP, where the mechanical and thermal withdrawal threshold of rats were significantly reduced. Mst1 was mildly detectable by Western blot in the sciatic nerve of rats in sham group, but became robust in the injured sciatic nerve after CCI. Mst1 siRNA applied to the injured sciatic nerve alleviated heat hyperalgesia and mechanical allodynia of rats, verifying a protective role of Mst1 knockdown in NP. The involvement of demyelination in the pathogenesis of neuropathic pain has been well established [26–28]. TEM and IF analysis of the injured sciatic nerve revealed myelin disruption and disorganization after CCI. And these disruptive effects were partly counteracted by Mst1 knockdown, suggesting that Mst1 knockdown might alleviated NP via myelin protection.

The involvement of mitochondrial damage in NP has been investigated recently [10, 29–32]. Parkin-dependent pathway has been suggested as one of the several main pathways of mitochondria priming for mitophagy. In response to mitochondrial damage, full length PTEN-induced kinase 1 (PINK1) is accumulated on the MOM [33], recruits Parkin from cytosol to mitochondria, and then activates its E3 ligase activity via phosphorylation [34]. Upon activation, Parkin ubiquitinates various proteins on the MOM leading to the recruitment of autophagy substrate, such as p62, which binds to ubiquitin-tagged MOM proteins to promote the removal of mitochondria via autophagy [35]. In the later stage, the linkage of p62 to ubiquitin on the mitochondria and lipidated LC3-II on the autophagosome provides a physical attachment point for the clearance of mitochondria [36]. In the present study, CCI induced autophagy activation, indicated by enhanced LC3-II protein and abundant autophagosomes in response to nerve injury. This was associated with a robust increase of p62 and Parkin, suggesting that autophagic influx was impaired after CCI. Consequently, the autophagic substrate was accumulated, and mitochondria could not be efficiently degraded by mitophagy. Taken together, this information indicated that the blocked mitophagy/autophagy degradation rather than impaired autophagy activation was involved in CCI-injured nerve. The results were different from several previous studies in diabetic cardiomyopathy and liver fatty disease, wherein mitophagy activation was impaired as evidenced by reduced Parkin and LC3-II after high glucose or fat insult [13, 14, 37]. The sciatic nerve in our research was in a condition lacking of oxygen and nutrition because of the constricted vessels nurturing the nerve, which may partially explain the different implications of Parkin and mitophagy activity in various disease settings [38]. What is more, the increase of Parkin and p62 caused by CCI was counteracted by Mst1 knockdown, indicating that Mst1 knockdown could favor successful clearance of mitophagy/autophagy substrate.

We further investigated the mechanisms underlying Mst1-mediated regulation on Parkin-dependent mitophagy and the resulting effects on mitochondrial function. Since SCs play a critical role in NP and myelin degeneration was reversed by Mst1 knockdown in the present study, a starvation model of RSC96 cells in vitro was used to partly mimic the injured SCs in CCI. Consistent to the in vivo data, starvation induced an increased level of Parkin in RSC96 cells which was abolished by Mst1 knockdown. Additionally, the expression of p62 and Tom20 (MOM marker) was reduced after Mst1 knockdown, verifying that Mst1 knockdown promoted the process of mitophagy/autophagy and the degradation of mitochondria. The expression of Parkin and p62 in cytosol and mitochondria was then quantified by Western blot, respectively. We found that Mst1 knockdown increased the localization of Parkin and p62 on mitochondria. These results indicated that in response to starvation, Mst1 knockdown promoted the recruitment of Parkin and p62 to mitochondria. This was consistent with a previous study on diabetic cardiomyopathy, where Mst1 inhibition by melatonin treatment activated Parkin translocation [37]. However, in what manner Mst1 promoted Parkin translocation is far from clear and deserves further investigation. In addition, the reduced Parkin and p62 in the whole cell and cytosol can be explained by the enhanced degradation of mitophagy/autophagy substrate. To define whether the enhanced degradation of them was beneficial, we performed a series of functional experiments. The results showed that Mst1 knockdown contributed to mitochondria homeostasis and cell survival, as evidenced by reduced cellular ROS level, enhanced mitochondrial membrane potential, and prohibited apoptosis.

Our research characterized Mst1 as a mediator of CCI-induced NP for the first time. Hopefully, this study may offer possibilities for discovering new therapies to NP. There were some limitations in the present study. First, we did not intervene both Mst1 expression and mitophagy activity simultaneously; thus, we did not directly prove whether Mst1 mediated NP by regulating mitophagy. Still, it was reasonable to conclude that Mst1-regulated mitophagy was closely related with the pathogenesis of NP, associated with improved mitochondrial function and myelin protection. Second, the present study focused on the effects of Mst1 knockdown in SCs, not in neurons. Nevertheless, since SCs and myelin protection are critical in the process of NP, our study illuminated an important part of the mechanisms underlying the novel effects of Mst1 in NP.

## 5. Conclusions

Altogether, our study illustrated that Mst1 knockdown attenuated NP, associated with mitophagy regulation via promoting the recruitment of Parkin to mitochondria in SCs, which resulted in improved mitochondrial function and myelin protection.

## Data Availability

The data that support the findings of this study are available on request from the corresponding authors.

## Abbreviations

SCs: Schwann cells
NP: Neuropathic pain
Mst1: Mammalian sterile 20-like kinase 1
CCI: Chronic constriction injury
MOM: Mitochondria outer membrane
PWT: Paw withdrawal threshold
PWL: Paw withdrawal latency.
MMP: Mitochondrial membrane potential.
